# Changes After Leptin Administration in Partial Lipodystrophy and Factors Associated With Hepatic and Metabolic Response

**DOI:** 10.1210/jendso/bvaf067

**Published:** 2025-05-07

**Authors:** Baris Akinci, Nevin Ajluni, Rasimcan Meral, Adam Hugh Neidert, Maria Foss Freitas, Donatella Gilio, Hari Conjeevaram, Elif Arioglu Oral

**Affiliations:** Caswell Diabetes Institute and Division of Metabolism, Endocrinology & Diabetes, University of Michigan, Ann Arbor, MI 48109, USA; Technological Research Program, Izmir Biomedicine and Genome Center & Depark, Dokuz Eylul University Health Campus, 35330 Izmir, Turkey; Caswell Diabetes Institute and Division of Metabolism, Endocrinology & Diabetes, University of Michigan, Ann Arbor, MI 48109, USA; Caswell Diabetes Institute and Division of Metabolism, Endocrinology & Diabetes, University of Michigan, Ann Arbor, MI 48109, USA; Caswell Diabetes Institute and Division of Metabolism, Endocrinology & Diabetes, University of Michigan, Ann Arbor, MI 48109, USA; Caswell Diabetes Institute and Division of Metabolism, Endocrinology & Diabetes, University of Michigan, Ann Arbor, MI 48109, USA; Caswell Diabetes Institute and Division of Metabolism, Endocrinology & Diabetes, University of Michigan, Ann Arbor, MI 48109, USA; Division of Gastroenterology, Department of Internal Medicine, University of Michigan, Ann Arbor, MI 48109, USA; Caswell Diabetes Institute and Division of Metabolism, Endocrinology & Diabetes, University of Michigan, Ann Arbor, MI 48109, USA

**Keywords:** leptin, partial lipodystrophy, MASH, response, incretins

## Abstract

**Context:**

Partial lipodystrophy (PL) is a rare disease characterized by selective loss of subcutaneous fat.

**Objective:**

To evaluate changes in apolipoproteins, hepatokines, hormones, appetite regulators, and inflammatory markers in patients with PL treated with leptin, assess postprandial metabolism and 24-hour dynamics, and identify predictors of hepatic and metabolic response to therapy.

**Methods:**

We studied 19 subjects from our previous clinical study (NCT01679197), which investigated the effect of leptin on metabolic dysfunction-associated steatohepatitis associated with PL. A mixed-meal test was performed in a subgroup of 14 patients, and paired 24-hour frequent sampling with standardized meals was completed in 5 individuals.

**Results:**

Leptin treatment led to reductions in apolipoproteins B, CII, CIII, and E (*P* < .05). Levels of ANGPTL3 tended to decrease after leptin treatment (*P* = .079). The mixed-meal test revealed significant reductions in triglyceride area under the curve (*P* = .017) and glucose excursions at several postmeal time points (*P* < .05). The immediate GIP secretion in response to a meal attenuated after leptin therapy (*P* = .005 at 60 minutes). Ghrelin levels showed an increase after leptin administration. The response to leptin treatment was associated with several factors, including baseline carbohydrate intake, leptin and triglyceride levels and triglyceride-rich apolipoproteins, and changes in triglyceride-rich apolipoproteins (*P* < .05 for all). Changes in IGF-1 levels were correlated with improvements in metabolic and liver parameters (*P* < .05).

**Conclusion:**

Leptin therapy modulates lipid metabolism, postprandial glucose regulation, and appetite signaling in patients with PL, with responses associated with metabolic parameters and carbohydrate intake.

Partial lipodystrophy (PL) syndromes are a heterogeneous group of diseases characterized by selective loss of body fat that can be inherited (familial partial lipodystrophy [FPLD]) or acquired (acquired partial lipodystrophy) [1-3]. Limited and dysfunctional peripheral fat depots along with relatively low levels of adipokines (such as leptin and adiponectin) in PL result in ectopic accumulation of fat and trigger the development of severe insulin resistance [4, 5].

PL is associated with multiple metabolic complications including diabetes, dyslipidemia, and metabolic dysfunction-associated steatotic liver disease (MASLD) [6-9]. Metabolic disease-associated steatohepatitis (MASH) (which was known as nonalcoholic steatohepatitis [NASH] at the time the study was conceived and funded) is a more severe hepatic disease associated with hepatocyte injury, inflammation, and fibrosis [10]. Hepatic inflammation caused by MASH can progress to cirrhosis and is now recognized as a precursor to cryptogenic cirrhosis [11]. This is 1 of the many risks of PL, which is now recognized as a complex multisystem disease driven by deficiency or dysfunction of adipose tissues [12, 13].

Recombinant human leptin (metreleptin) has been approved for the treatment of leptin deficiency and resultant complications in generalized lipodystrophy (GL) but is not yet approved for the treatment of PL in the United States, even though it is approved for patients with PL who remain inadequately controlled on typical lipid and glucose-lowering therapies in Europe [14]. The effect of leptin therapy on metabolic status in patients with GL has been robust, with significant improvements in glycated hemoglobin (HbA1c), triglyceride levels, and liver parameters [15, 16]. Exogenous leptin treatment has also shown benefits for metabolic disease in patients with PL, though these effects can be variable, with certain subgroups showing more pronounced improvements [17].

We have previously reported key findings from a clinical study [18] investigating the efficacy of leptin therapy in MASH associated with PL. Overall, we observed improvements in MASH parameters and triglyceride levels in the treatment group, although not all participants responded. Briefly, among 19 participants treated with exogenous leptin for 12 months, there was a significant reduction in fasting triglycerides, liver enzymes (alanine aminotransferase and aspartate aminotransferase), and resting energy expenditure. A slight, nonsignificant decrease in weight was also observed. Additionally, reductions in total food intake, total energy intake, fat, carbohydrate, and protein intake were noted, although these changes were not statistically significant. In 18 participants with paired liver biopsies, both NASH and the nonalcoholic fatty liver disease activity score (NAS) showed significant improvement. Fasting glucose and HbA1c levels also tended to decrease following treatment.

Another interesting observation was that leptin therapy led to the attenuation of circulating glucose insulinotropic peptide (GIP) levels and an increase in ghrelin levels in individuals with MASH who had no formal diagnosis of lipodystrophy but relative leptin deficiency [18]. Leptin regulates appetite by acting on the hypothalamus in the brain, where it binds to leptin receptors and inhibits orexigenic neurons, such as those expressing neuropeptide Y and agouti-related peptide, while activating anorexigenic (appetite-suppressing) neurons that produce pro-opiomelanocortin [19]. Leptin is also known to interact with incretin hormones, such as glucagon-like peptide-1 (GLP-1) and GIP, which play key roles in glucose metabolism and appetite regulation. Additionally, GLP-1 and leptin share overlapping pathways in the hypothalamus, where they synergistically reduce food intake [20].

It has been previously shown that levels of triglyceride-rich lipoprotein particles are increased in lipodystrophy and that this atherogenic lipid profile improves after exogenous leptin therapy [21]. Also, baseline triglyceride levels seem to be an important predictor of clinical response to leptin therapy in PL with patients with more severely elevated triglycerides tend to respond better [17]. To further elucidate these changes in a heterogeneous PL cohort and study their association with treatment response, we measured levels of a wide range of apolipoproteins and also angiopoietin-like protein 3 (ANGPTL3) and angiopoietin-like protein 4 (ANGPTL4) at baseline and at different time points after exogenous leptin therapy. ANGPTL3 and ANGPTL4 are glycoproteins that regulate triglyceride metabolism by inhibiting lipoprotein lipase. ANGPTL3 is primarily expressed in the liver and is elevated in leptin-deficient states, but leptin administration reduces its expression [22]. Previous research has shown that ANGPTL3 levels are elevated in patients with GL and decrease with leptin replacement therapy [23]. Given their metabolic role, we hypothesized that studying ANGPTL3 and ANGPTL4 levels in our heterogeneous PL cohort would be of interest, particularly to assess their changes following exogenous leptin treatment.

Insulin stimulates the hepatic expression of insulin-like growth factor 1 (IGF-1) and its binding protein IGF-binding protein (IGFBP)-3, largely through activation of the PI3K-AKT signaling pathway. In insulin-resistant states, impaired hepatic insulin signaling leads to reduced IGF-1 synthesis [24]. Low IGF-1 levels contribute to metabolic dysfunction, as IGF-1 has insulin-sensitizing and anti-inflammatory properties that protect against hepatic steatosis and fibrosis [25]. In contrast, improvements in insulin resistance and liver health could improve the liver's responsiveness to GH, promoting IGF-1 secretion. Given the role of IGF-1 in metabolic regulation, we hypothesize that improvements in insulin sensitivity and liver histopathology following exogenous leptin therapy would be associated with changes in IGF-1 levels, which may correlate with improvements in metabolic disease.

In this study, we aimed to (1) determine changes in circulating apolipoprotein concentrations, hepatokines, hormones, appetite regulators, and inflammation markers in patients with PL who have been treated with leptin; (2) evaluate postprandial insulin and incretin secretion, along with appetite regulator levels, triglycerides, and free fatty acids (FFAs); (3) investigate 24-hour changes in leptin, incretins, and appetite regulators after acute and chronic administration of leptin; and (4) identify predictors of hepatic and metabolic responses. For this purpose, we analyzed samples and performed a responder analysis in patients treated with leptin therapy as part of our previously reported study.

## Methods

### The Parent PL Study

A detailed description of this open-label study performed at the University of Michigan, Ann Arbor, MI, was previously published [12, 18]. The study was funded by the National Institute of Diabetes and Digestive and Kidney Diseases (NIDDK) and approved by the University of Michigan IRBMED (ClinicalTrials.gov identifier: NCT01679197, IRBMED: HUM00058708); all participants gave informed consent.

Patients enrolled in the study had the diagnosis of acquired or inherited PL made by physician assessment. The presence of liver disease was assessed by ultrasound or prior liver biopsy demonstrating fatty liver disease. Exclusion criteria included presence of human immunodeficiency virus, advanced liver disease (laboratory values demonstrating abnormal synthetic function), viral hepatitis, alcohol consumption greater than 40 g per week, end-stage renal disease, active cancer, greater than New York Heart Association class 2 congestive heart failure, known allergy to *Escherichia coli*-derived proteins or hypersensitivity to any component of metreleptin treatment. Informed consent was obtained from all patients or guardians. The first patient was enrolled on October 8, 2012, and data collection for this study was completed on April 14, 2016.

The primary outcome was the global NASH score from liver biopsy, while secondary outcomes included changes in hepatic fat percentage, HbA1c, lipid levels, and energy expenditure with metreleptin therapy.

The starting dose of metreleptin was 2.5 mg daily in the male subjects and 5 mg in the female subjects. Average metreleptin dose used per subject across the study was 7.04 ± 1.99 mg/day. The maximum dose was 10 mg/day. Metabolic therapy was kept stable except for necessary down-titration to prevent hypoglycemia. Minor increases in glucose-lowering treatments occurred in a few cases, with detailed treatment changes reported previously.

The main results of the PL study were previously reported [18].

### Current Study Cohort and Substudy Populations

Because we had previously observed a change in circulating GIP and ghrelin levels in nonlipodystrophic individuals with MASH and low leptin levels treated with exogenous leptin, we had a priori made decision to interrogate incretin levels as well as other hormones that may play a role in regulating food intake and lipid metabolism. From the parent protocol, 14 participants were assigned to a mixed-meal schema and 8 individuals were assigned to the frequent blood sampling schema (paired sampling available in 5 subjects) based on participant volition on a first-come, first-choice basis.

**Figure 1.**
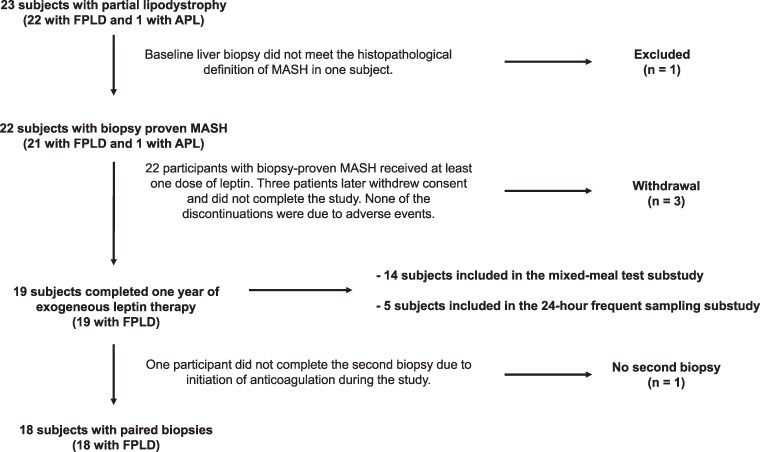
Study diagram. Abbreviations: APL, acquired partial lipodystrophy; FPLD, familial partial lipodystrophy; MASH, metabolic disease-associated steatohepatitis.

A study diagram is presented in Fig. 1. Twenty-three participants with PL were enrolled and completed baseline study procedures. One patient was excluded because her baseline liver biopsy did not meet the histopathological definition of MASH. The remaining 22 participants had biopsy-proven MASH and continued the protocol, receiving at least 1 dose of metreleptin. Of 22 subjects, 19 completed 12 months of exogenous leptin treatment (mean age: 43 ± 17 years, range: 12-64; 16 females and 3 males), and 18 completed a second liver biopsy (mean age: 43 ± 17 years, range: 12-64; 15 females and 3 males). One participant did not complete the second biopsy due to initiation of anticoagulation during the study.

Of the 22 patients enrolled, 14 were included in the mixed-meal substudy and 8 were initiated on the frequent sampling substudy. There were 14 completers and 13 with paired biopsies in the mixed-meal substudy. There were 5 who completed the study in the frequent sampling substudy. Table 1 presents the baseline characteristics of the overall study cohort and the substudy populations. Individual level data are shown in Table 2.

**Table 1.** Baseline characteristics of the overall study population and the substudy populations

	Study population (n = 19)	Mixed-meal test substudy population (n = 14)	Frequent sampling substudy population (n = 5)	*P* value
Age (y)	43 ± 17	44 ± 16	41 ± 20	.929
Gender (M:F)	16:3	11:3	5:0	.529
Pathologic *LMNA* variant (n, %)	6 (32%)	3 (21%)	3 (60%)	.281
BMI (kg/m^2^)	26.8 ± 5.8	26.9 ± 6.3	26.5 ± 4.5	.992
Body weight (kg)	76 ± 22	77 ± 24	71 ± 14	.836
Waist/hip ratio	1.00 ± 0.08	1.01 ± 0.8	0.99 ± 0.07	.878
FMR	1.76 ± 0.54	1.59 ± 0.24	2.23 ± 0.88	.072
Glucose (mg/dL)	193 ± 84	211 ± 91	142 ± 28	.291
Hemoglobin A1c (%)	8.8 ± 1.8	9.0 ± 1.9	8.3 ± 1.6	.759
Triglyceride (mg/dL)*^a^*	1244 ± 1873	1487 ± 2114	563 ± 686	.604
Total cholesterol (mg/dL)	268 ± 147	278 ± 163	239 ± 100	.882
LDL cholesterol (mg/dL)	89 ± 47	80 ± 38	115 ± 64	.353
HDL cholesterol (mg/dL)	34 ± 10	31 ± 7	43 ± 12	.051
Fasting leptin level (ng/mL)	20.9 ± 14.1	16.4 ± 9.3	33.4 ± 18.5	.059
ALT (IU/L)*^a^*	51 ± 33	49 ± 27	57 ± 49	.995
AST (IU/L)*^a^*	42 ± 29	42 ± 31	42 ± 28	.998
NASH score	6 ± 2	6 ± 2	6 ± 1	.650
NAS	5 ± 1	5 ± 1	4 ± 1	.761

Categorical parameters are compared using the chi-square test. ANOVA is used to compare continuous variables.

Abbreviations: ALT, alanine aminotransferase; AST, aspartate aminotransferase; BMI, body mass index; FMR, fat mass ratio (%fat trunk/%fat legs); HDL, high-density lipoprotein; LDL, low-density lipoprotein; *LMNA*, lamin A/C; NASH score, the sum of scores for steatosis, lobular inflammation, ballooning and fibrosis; NAS, nonalcoholic fatty liver disease activity score, the sum of scores for steatosis, lobular inflammation, and ballooning.

^a^Tests are run on log-transformed data. Data are presented as mean ± standard deviation (SD) or frequency.

**Table 2.** Individual characteristics of the study cohort

Age	Gender	LD type	Genetic variant	BMI (kg/m^2^)	Leptin (ng/mL)
39	F	FPLD	*FBN1* p.M1576T*^a^*	31.2	16.9
55	M	FPLD	None identified	28.2	13.4
53	F	FPLD	None identified	35.9	34.1
64	M	FPLD	None identified	35.2	24.4
34	F	FPLD	*POLD1* p.E1067K	18.1	9.0
14	F	FPLD	*POLD1* p.E1067K	19.0	11.5
58	F	FPLD	None identified	31.0	26.5
54	F	FPLD	None identified	24.1	10.5
33	F	FPLD	*LMNA* p.R60G	19.8	16.6
34	M	FPLD	None identified	34.0	19.1
63	F	FPLD	*DYRK1B* p.S462R*^a^*	30.9	48.9
57	F	FPLD	*LMNA* p.R482Q	24.9	16.2
12	F	FPLD	None identified	17.7	7.2
57	F	FPLD	None identified	30.3	35.2
42	F	FPLD	*LMNA* p.R482Q	24.0	6.4
59	F	FPLD	*LMNA* p.R482Q	21.9	11.8
41	F	FPLD	*LMNA* p.R482W	25.9	12.8
37	F	FPLD	*LMNA* p.R349W	26.4	55.4
13	F	FPLD	None identified	30.5	35.6

Abbreviations: BMI, body mass index; *DYRK1B*, dual-specificity tyrosine phosphorylation-regulated kinase 1b; *FBN-1*, fibrillin-1; FPLD, familial partial lipodystrophy; LD, lipodystrophy; *LMNA*, lamin A/C; *POLD1*, polymerase (DNA-directed), delta 1, catalytic subunit-1.

^a^Variant of uncertain significance. Individual patient characteristics were previously published [18].

### Intervention Period

One week before the first visit, participants were contacted by the study dietitian to begin a standardized diet. This diet was well-balanced (50% carbohydrate, 20% protein, and 30% fat) consisting of a calculated weight maintenance calories per day. Alcohol intake was limited to 1 serving of alcoholic drink per week and participants were asked to avoid alcohol consumption for 72 hours before scheduled study visits. They were instructed to consume a standardized dinner meal prior to the evening before study visits (750 calories, same composition as previously noted).

Participants were admitted to the Michigan Clinical Research Unit on day 1 to obtain baseline assessments of follow-up study procedures. Following baseline study procedures, participants began exogenous leptin. Metreleptin was provided by Amylin Pharmaceuticals (San Diego, CA), later manufactured by Bristol Myers Squibb and Aegerion Pharmaceuticals, and then Amryt Pharmaceuticals, which was recently acquired by Chiesi Farmaceutici. Participants self-administered subcutaneous injections after reconstitution once a day. Metreleptin was started at a dose of 2.5 mg per day in males and 5 mg per day in females. The dose was increased to a maximum of 10 mg daily (in single or divided doses based on individual's preference) after 3 months of the starting dose at the investigator's discretion to achieve improved glucose and triglyceride control.

### Mixed-meal Test Substudy Schema

Participants assigned to this substudy ingested 474 mL of Optifast (320 kcal, 50% carbohydrate, 35% protein, 15% fat) and blood was collected in regular red top tubes for measurement of glucose, insulin, free fatty acid levels, and triglycerides. In addition, a 10-mL blood sample was collected in tubes containing Pefabloc SC (Sigma-Aldrich, St. Louis, MO) and DPP-IV inhibitor (EMD Millipore, Billerica, MA) for determination incretin measurements at baseline and after 30, 60, 90, 120, and 180 minutes. The study was conducted at baseline before initiation of therapy and 6 months and 12 months of therapy.

### Frequent Sampling Substudy Schema

Participants in this substudy were admitted for an additional 24 hours before treatment was initiated. On the day of testing, they were given a daily caloric intake of weight maintenance with 50% carbohydrate (complex carbohydrates), 20% lean protein, and 30% healthy fats prepared by Michigan Metabolic Kitchen. The calories were proportioned into three meals served at 8 Am, 12 Pm, and 6 Pm. Meals were prepared considering participant preferences as well. Participants were asked to eat all of the food. Trays were controlled by dietitian team. Blood samples were obtained for 24 hours, sampled at 30-minute intervals using an antecubital catheter. A sample was skipped if there was more than 15 minutes of delay in case of lost IV access. Sampling was carried out at baseline on the day of initiation of therapy and at 12 months, with full daily dose given at 8 Am.

### Assessment of Liver Fat and Histopathology

Even though the methods were previously described [18], it is useful to restate how liver health was assessed in this study. To evaluate hepatic fat content, magnetic resonance imaging (MRI) using quantitative multi-echo Dixon method and multi-echo MR spectroscopy was used; this method has been described previously [18]. Histopathological grading for global NAS and NASH scores was performed on hematoxylin and eosin and trichrome stained slides of liver biopsies. Paired liver biopsies were performed at baseline, before the initiation of leptin therapy, and one year after treatment. The primary outcome was the global NASH score. Changes in individual components of the NASH and fibrosis scores were evaluated in a blinded manner to assess the impact of metreleptin on liver histopathology. The scores were determined by histopathological examination using the simplified clinical criteria, which is a modified and clinically applied version of National Institutes of Health (NIH) NASH Clinical Network Criteria [26]. Global NASH score equals the sum of scores for steatosis (0-3), lobular inflammation (0-3), hepatocellular ballooning (0-2), and fibrosis (0-4). NAS includes all these parameters except fibrosis. The definitive diagnosis of MASH on liver histopathology requires the presence of at least steatosis, lobular inflammation, and hepatocellular ballooning [10]. Only subjects meeting the clinical diagnosis of MASH were advanced on the protocol. Liver biopsy was repeated at the 12-month visit. Changes in the components of the NAS score and fibrosis score were assessed in a blinded fashion after 1 year of exogenous leptin treatment.

### Definition of Responders

A hepatic response was defined as a decrease in the total NASH score of ≥2 without an increase in fibrosis. Glycemic response was defined as a reduction in HbA1c of >1% from baseline. Lipid response was defined as a decrease of >30% in triglycerides from baseline. Patients with any 2 of the 3 criteria at month 12 were considered to have shown a significant metabolic response to metreleptin therapy.

### Study Measurements

#### Assessment of body composition

Body composition was evaluated using skin thickness measurements and waist and hip circumferences using standardized techniques. Dual-energy X-ray absorptiometry (DEXA) (GE Lunar Prodigy, model PA +41744, Madison, WI) was used to estimate fat and lean body mass. Data from DEXA evaluation was used to calculate FMR, a ratio of the percentage of the trunk fat mass to the percentage of the lower limb fat mass. Fat shadow images were generated by processing the DEXA scan files (.dfb) and analyzed by using enCore v14.10 as described previously [27].

#### Energy intake and resting energy expenditure

Energy intake was assessed using 3-day food records. Subjects were asked to record the type and amount of food and beverage consumed on 2 consecutive weekdays and 1 weekend day using standardized measures. Records were reviewed by study dietitians. Food intake data were analyzed and energy and nutrient intake were calculated using the Nutritionist V Diet Analysis software (First DataBank, Inc., San Bruno, CA). Resting energy expenditure (REE) was measured after 30 minutes of rest using a microprocessor-controlled indirect calorimetry device (Sensormedics, Yorba Linda, CA) that measures oxygen consumption and carbon dioxide production [28].

#### Biochemistry

Blood samples were collected for measurement of all analytes after a 10-hour fast. For measurement of incretin levels, samples were collected in tubes containing Pefabloc SC (Sigma-Aldrich, St. Louis, MO) and DPP-IV inhibitor (EMD Millipore, Billerica, MA). Fasting biochemistry laboratory values were determined in the Clinical Pathology Laboratory of the University of Michigan using auto-analyzer equipment. Leptin levels were analyzed using commercial ELISA (EMD Millipore, Billerica, MA). Other cytokines were measured with the Quantikine ELISA (R&D Systems, Minneapolis, MN).

### Statistical Methods

Statistical analyses were performed using GraphPad Prism version 8 (La Jolla, CA) and SPSS version 22.0 (SPSS, Inc., Chicago, IL, USA). The sample size was too small in the frequent sampling substudy. Their baseline characteristics were compared with those of the overall study population and the mixed-meal substudy population; otherwise, no formal statistical analyses were performed on this substudy population. Categorical parameters were compared using the chi-squared test. An independent sample *t*-test was used to compare continuous variables. ANOVA was used to determine differences between 3 groups. The repeated-measures ANOVA was used to compare variables that were based on repeated observations. Tukey's test was used as a post hoc test. Paired *t*-test was used to compare month 12 values to baseline as the change at 12 months vs baseline was a prespecified endpoint. Log transformation was used for skewed data. Correlations were investigated by calculating Pearson's correlation coefficient. Received operating characteristic (ROC) analysis was used to determine potential predictors of exogenous leptin response. Data were presented as mean ± standard deviation (SD) or frequency unless otherwise stated. Two-tailed *P* value was calculated, and *P* < .05 was considered statistically significant.

## Results

### Levels of Apolipoproteins, Hepatokines, Hormones, Inflammation Markers, and Appetite Regulators After Exogenous Leptin Treatment

**Figure 2.**
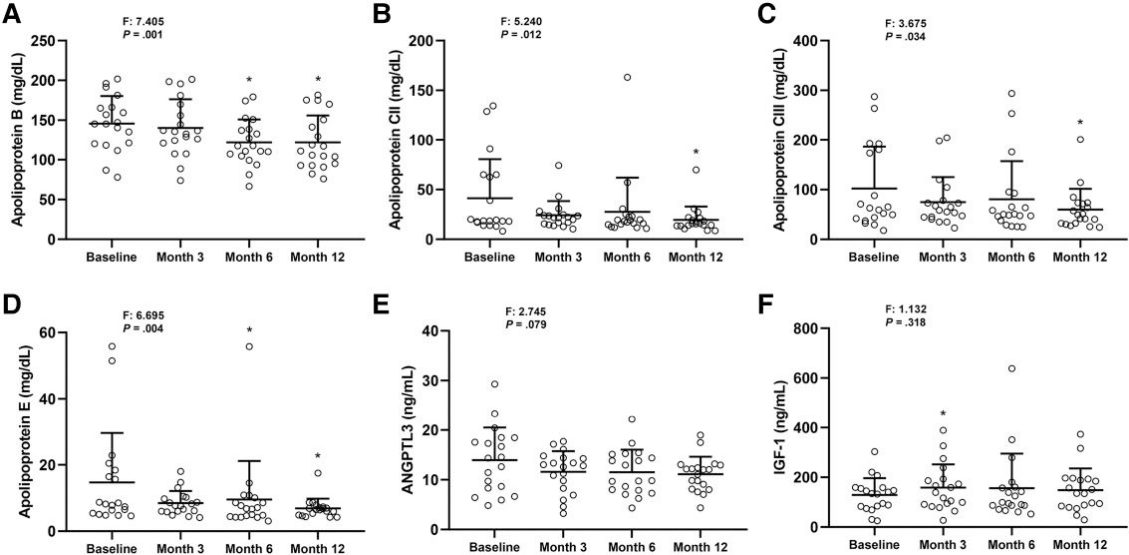
Levels of apolipoproteins, ANGPTL3, and IGF-1 over the 12-month treatment period with exogenous leptin. Levels of apolipoprotein B (A), apolipoprotein CII (B), apolipoprotein CIII (C), apolipoprotein E (D), ANGPTL3 (E), and IGF-1 (F) in subjects with partial lipodystrophy treated with metreleptin for 1 year. The F-statistic and *P* value are reported from a repeated-measures ANOVA. *Adjusted *P* < .05 vs baseline (Tukey post hoc test). Paired *t*-test was used to compare month 12 values to baseline (without multiplicity correction) as the change at 12 months vs baseline was a prespecified endpoint. Tests are run on log-transformed data for apolipoprotein CII, apolipoprotein CIII, and apolipoprotein E. The data are reported as mean ± SD.

Levels of apolipoproteins B (Fig. 2A), CII (Fig. 2B), CIII (Fig. 2C), and E (Fig. 2D) decreased significantly throughout the treatment. These changes were significant at month 12. Levels of ANGPTL3 also tended to decrease after exogenous leptin (Fig. 2E). ANGPTL3 levels decreased from 14 ± 7 ng/mL to 12 ± 4 ng/mL at month 3, 12 ± 5 ng/mL at month 6, and 11 ± 4 ng/mL at month 12. The percent reduction at month 12 was 6% ± 42%, with limited clinical relevance. There were no statistically significant changes in apolipoprotein AI, apolipoprotein AII, and ANGPTL4 levels (data not shown). IGF-1 levels significantly increased at month 3 (158 ± 94 ng/mL vs 129 ± 68 ng/mL at baseline, *P* = .019); however, the difference was not significant over time (Fig. 2F). We also measured IGFBP-1, IGFBP-2, and IGFBP-3 at baseline and 3 months, but these changes were not significant (data not shown). Fasting morning levels of ghrelin, GLP-1, GIP, peptide YY, free triiodothyronine, free thyroxine, TSH, cortisol, IL-1, and IL-6 were not significantly different than baseline at the end of the study (data not shown); however, reverse free triiodothyronine levels trended to decrease at month 12 (22.5 ± 4.0 ng/dL vs 86.9 ± 115.7 ng/dL, *P* = .085).

### Mixed-meal Test Substudy

**Figure 3.**
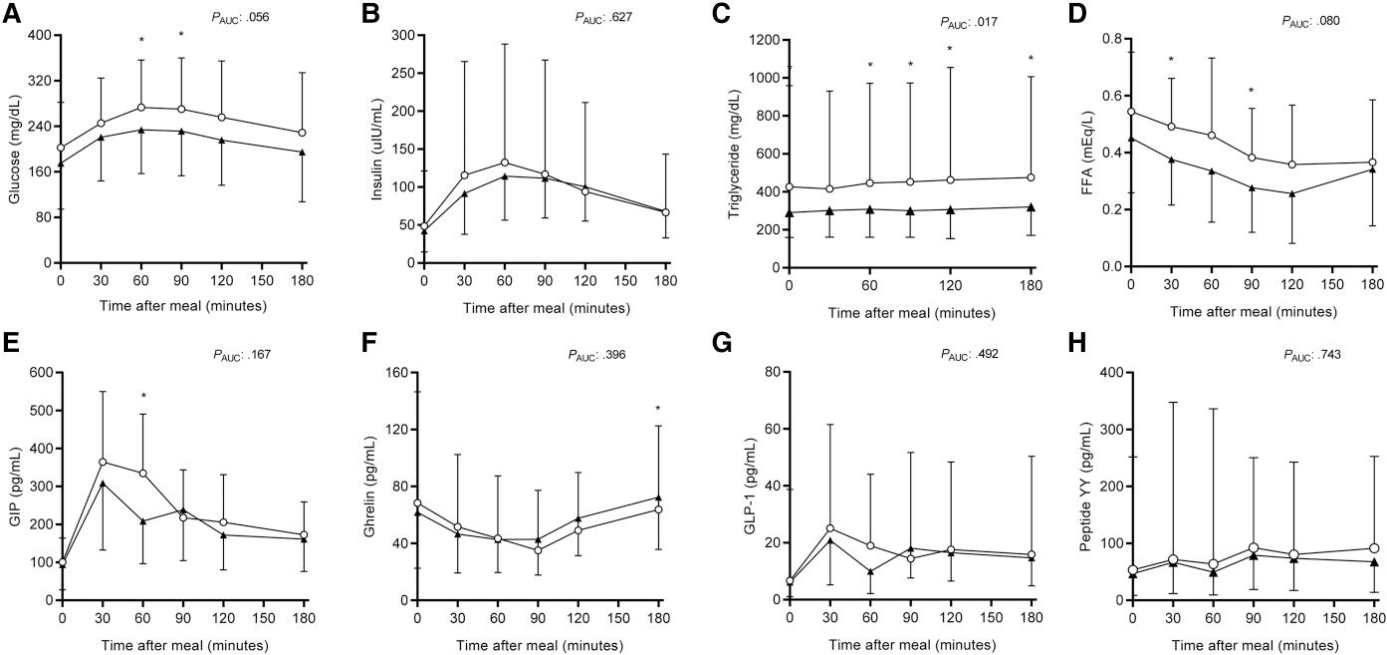
Mixed-meal testing results. A subset of subjects (n = 14) underwent mixed-meal testing. Levels of (A) glucose, (B) insulin, (C) triglycerides, (D) free fatty acids (FFAs), (E) GIP, (F) ghrelin, (G) GLP-1, and (H) peptide YY are presented at baseline ○ and following 12 months of metreleptin therapy ▴. The values are presented as mean ± SD if data of all time points show a normal distribution (glucose, FFAs, GIP, and ghrelin). Otherwise, the data are presented as geometric mean ± geometric SD (insulin, triglycerides, GLP-1, and peptide YY). *P*_AUC_ values are shown for comparisons of the area under the curve (AUC). Specific time point comparisons are denoted with * if *P* < .05 at the specific time point. Abbreviations: FFA, free fatty acids; GIP, glucose insulinotropic peptide; GLP-1, glucagon-like peptide.

In this substudy, we investigated metabolic and hormonal response to mixed-meal test in 14 participants. In both pre- and postexogenous leptin therapy tests, glucose and insulin levels increased after mixed-meal, reaching their peak levels at 60-minutes after mixed-meal (glucose: F: 18.793, *P* < .001 before leptin, and F: 21.666, *P* < .001 after leptin; insulin: F: 13.647, *P* < .001 before leptin, and F: 11.643, *P* < .001 after leptin). Postmeal glucose excursion was attenuated after 12 months of leptin therapy at 60 minutes (*P* = .043) and 90 minutes (*P* = .032). The glucose area under the curve (AUC) tended to decrease (Fig. 3A, *P* = .056). There was no significant difference in postmeal insulin (Fig. 3B, *P* = .627) but 8 of 14 participants were on insulin with 4 of them being on U-500 insulin. Percent increases from baseline levels were not significant in glucose and insulin values before and after exogenous leptin treatment (*P* > .05).

Triglyceride levels showed a slight increase from baseline to post-meal before leptin (F: 4.511, *P* = .008), which was attenuated after treatment (F: 1.881, *P* = .174). Triglyceride AUC was significantly lowered after leptin therapy (Fig. 3C, *P* = .017). Fasting and 60-, 90-, 120-, and 180- minute triglyceride levels were also significantly decreased (*P* = .025, *P* = .026, *P* = .010, *P* = .029, and *P* = .014, respectively). Fasting FFAs showed suppression after the mixed-meal (F: 6.214, *P* = .004 before leptin, and F: 10.638, *P* < .001 after leptin). The maximum suppression was observed at 120 minutes postmeal (Fig. 3D). After leptin therapy, FFAs were lowered compared to before treatment levels as well as those measured at 30 and 90 minutes (*P* = .030 and *P* = .037, respectively), although the difference in FFA AUC was not statistically significant (*P* = .080). Similarly, percent suppression from baseline levels was not significantly different before and after leptin (*P* > .05).

GIP levels increased and reached its peak level at 30 minutes during the mixed-meal (F: 16.125, *P* < .001 before leptin, and F: 9.456, *P* < .001 after leptin). GIP levels measured at 60 minutes (Fig. 3E, *P* = .005) were decreased, but not at other time points. Also, percent increase from baseline was attenuated after treatment with leptin (369 ± 301% vs 172 ± 141%, *P* = .034). Ghrelin levels showed a reduction reaching the lowest levels at 90 minutes (F: 5.763, *P* = .012 before leptin, and F: 3.639, *P* = .034 after leptin). Postmeal ghrelin levels were increased at 180 minutes (*P* = .007); however, the differences were not significant in ghrelin AUC (Fig. 3F, *P* = .396) or at other time points. Although GLP-1 levels increased after the meal (GLP-1: F: 8.546, *P* < .001 before leptin, and F: 3.159, *P* = .043 after exogenous leptin; Fig. 3G), the levels did not change significantly before and after treatment. There were no significant changes in pre- or postmeal peptide YY (Fig. 3H). Changes at month 6 were generally similar, with less notable decreases in triglycerides and FFAs (data not shown).

### 24-hour Frequent Sampling Substudy

**Figure 4.**
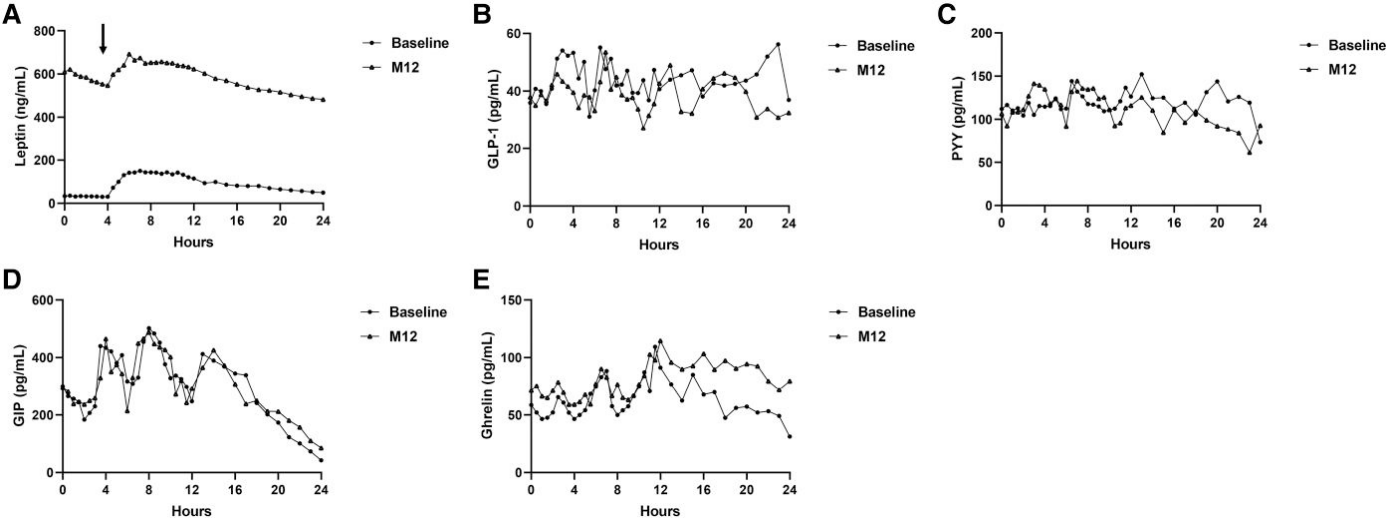
A 24-hour frequent sampling substudy. The effects of acute and chronic leptin administration on serum leptin (A), peptide YY (B), GLP-1 (C), GIP (D), and ghrelin (E) were analyzed in 5 individuals on the first day of treatment initiation and 1 year after leptin treatment. The arrow indicates the timing of leptin injection. Abbreviations: GIP, glucose insulinotropic peptide; GLP-1, glucagon-like peptide; PYY, peptide YY.

We studied the effects of acute and chronic leptin administration on serum leptin levels, incretin hormones, and appetite regulators in 5 individuals during the first day of treatment initiation and 1 year after leptin treatment. Following SC bolus dosing (at hour 4), serum leptin levels increased in parallel with the absorption of metreleptin, reaching peak levels approximately 2.5 to 3 hours after injection and plateauing for about 4 to 5 hours (Fig. 4A). These levels then gradually decreased over time. We observed significantly higher leptin concentrations in subjects after 1 year of leptin treatment; however, postinjection kinetics remained similar. The higher levels could be attributed to chronic treatment with metreleptin. However, it is important to note that anti-leptin antibodies, which develop in most patients after metreleptin therapy, can interfere with the measurement of plasma leptin concentrations by competing with the assay's capture and detection antibodies [29].

Although fluctuations were observed, we did not detect a significant change in GLP-1 and peptide YY after leptin administration (Fig. 4B and 4C). GIP levels increased after meal ingestion. The trends were similar after acute or chronic leptin administrations (Fig. 4D). Ghrelin levels showed an increase after leptin administration. This ghrelin increase was relatively short lasting after acute leptin administration (Fig. 4E).

### Baseline Levels of Apolipoproteins and ANGPTL3 are Correlated With Metabolic Status

Table 3 shows correlation coefficients. At baseline, levels of apolipoprotein B tended to correlate with total cholesterol and low-density lipoprotein (LDL)-cholesterol. Apolipoproteins CII, CIII, and E were negatively correlated with body mass index (BMI) and positively correlated with total cholesterol and triglycerides. Apolipoprotein CII was positively correlated with HbA1c, and apolipoproteins CIII and E tended to correlate with HbA1c. Apolipoprotein CII was correlated with liver fat on MRI and tended to correlate with NAS. Apolipoprotein CIII tended to correlate with liver fat. Apolipoprotein E was correlated with NAS and tended to correlate with AST. Apolipoprotein AI and AII concentrations were positively correlated with high-density lipoprotein cholesterol and negatively correlated with carbohydrate intake. There was also a positive correlation between apolipoprotein AI and LDL cholesterol. ANGPTL3 levels were positively correlated with total cholesterol, triglycerides, and apolipoproteins CII, CIII, and E, and fasting GLP-1. ANGPTL4 was also positively correlated with GLP-1 and tended to correlate with HbA1c.

**Table 3.** Correlation analysis of baseline apolipoproteins, ANGPTL3, ANGPTL4, leptin, and the studied parameters

	Apo B	Apo CII	Apo CIII	Apo E	Apo AI	Apo AII	ANGPTL3	ANGPTL4	Leptin
BMI, *r* *P* value	NS	-.484	-.496	-.537	NS	NS	NS	NS	.521
		**.036**	**.031**	**.018**					**.022**
Waist/hip ratio, *r* *P* value	NS	NS	NS	NS	NS	NS	NS	.414	.533
								.078	**.019**
Total cholesterol, *r* *P* value	.449	.861	.787	.896	NS	NS	.668	NS	NS
	.054	**<.001**	**<.001**	**<.001**			**.002**		
LDL cholesterol, *r* *P* value	.415	-.482	-.470	-.448	.512	NS	NS	NS	.490
	.077	**.037**	**.042**	.054	**.025**				**.033**
HDL cholesterol, *r* *P* value	NS	NS	NS	NS	.485	.488	NS	NS	.597
					**.035**	**.034**			**.007**
Triglyceride, *r* *P* value	NS	.945	.944	.954	NS	NS	.709	NS	-.463
		**<.001**	**<.001**	**<.001**			**.001**		**.046**
HbA1c, *r* *P* value	NS	.471	.400	NS	NS	NS	NS	.419	NS
		**.042**	.090					.074	
AST, *r* *P* value	NS	NS	NS	.421	NS	NS	NS	NS	NS
				.072					
Liver fat on MRI, *r* *P* value	NS	.469	.415	NS	NS	NS	NS	NS	NS
		**.043**	.077						
NAS, *r* *P* value	NS	.433	NS	.471	NS	NS	NS	NS	-.543
		.064		**.042**					**.016**
Carbohydrate intake, *r* *P* value	NS	NS	NS	NS	-.503	-.470	NS	NS	NS
					**.033**	**.049**			
Apo CII, *r* *P* value	.411	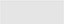	.950	.956	NS	NS	.664	NS	-.449
	.080		**<.001**	**<.001**			**.002**		.054
Apo CIII, *r* *P* value	NS	.950	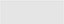	.926	NS	NS	.664	NS	-.416
		**<.001**		**<.001**			**.002**		.076
Apo E, *r* *P* value	NS	.956	.926	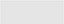	NS	NS	.667	NS	−.447
		**<.001**	**<.001**				**.001**		.055
Apo AII, *r* *P* value	NS	NS	NS	NS	.393	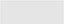	NS	NS	.435
					.096				.063
GLP-1, *r* *P* value	NS	NS	NS	NS	NS	NS	.491	.584	NS
							**.033**	**.009**	

Bold values denote statistical significance at the *P* < .05 level.

Abbreviations: ANGPTL3, angiopoietin-like protein 3; ANGPTL4, angiopoietin-like protein 4; Apo, apolipoprotein; AST, aspartate aminotransferase; BMI, body mass index; GLP-1, glucagon-like peptide-1; HDL, high-density lipoprotein; LDL, low-density lipoprotein; MRI, magnetic resonance imaging; NAS, nonalcoholic fatty liver disease activity score, the sum of scores for steatosis, lobular inflammation, and ballooning.

### Baseline Levels of Triglyceride, Apolipoproteins, and ANGPTL3 Correlates With Changes After Exogenous Leptin Treatment

**Figure 5.**
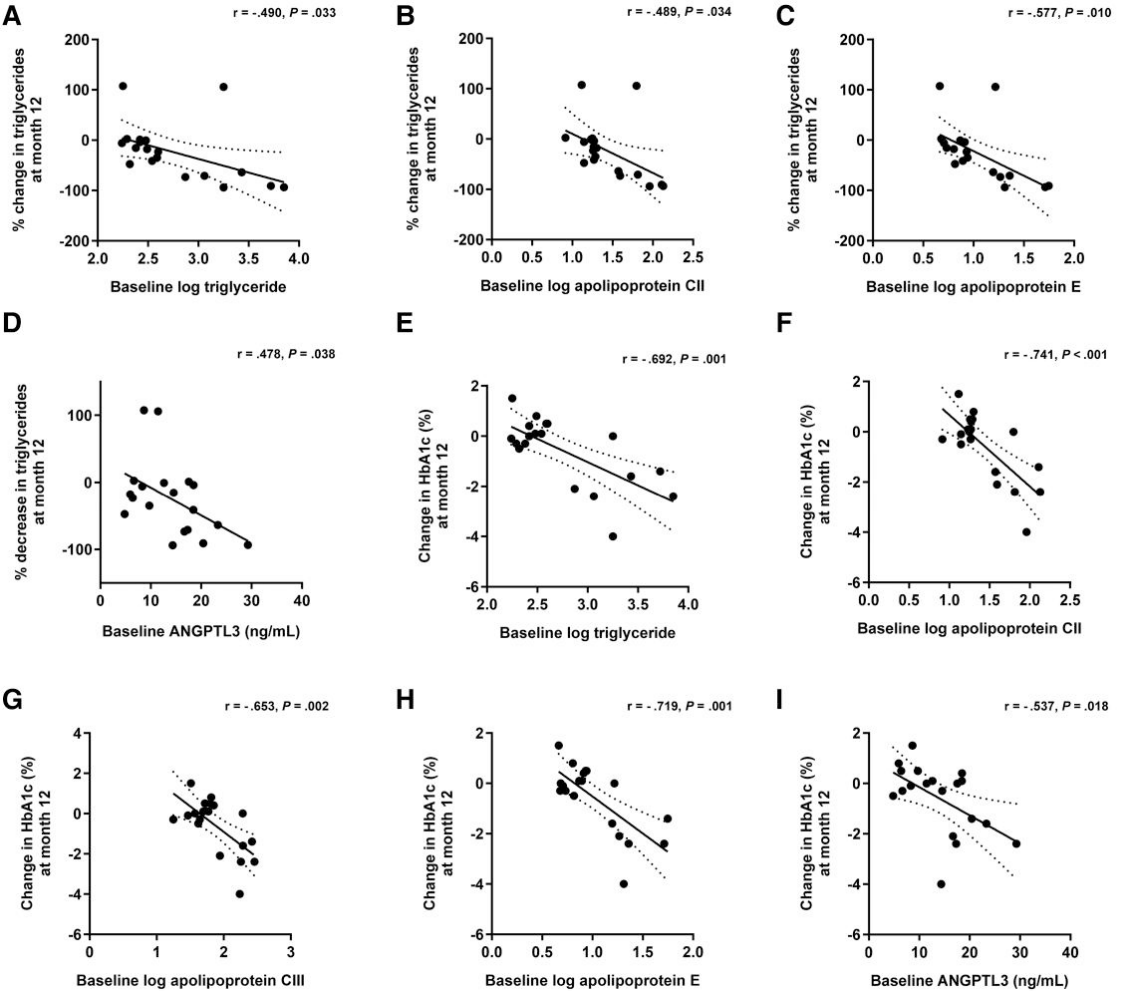
Correlation of baseline triglycerides, HbA1c, and levels of apolipoproteins and ANGPTL3 with changes in triglycerides and HbA1c after treatment with exogenous leptin. Correlation of baseline triglycerides with changes in triglycerides (A) after treatment with exogenous leptin. Correlation of baseline levels of apolipoproteins CII (B) and E (C), and ANGPTL3 (D) with percent changes in triglycerides after treatment with exogenous leptin. Correlation of baseline triglycerides (E), and apolipoproteins CII (F), CIII (G), and E (H), and ANGPTL3 (I) with changes in HbA1c.

Baseline triglyceride levels were correlated with percent (Fig. 5A) and numeric changes (*r* = -.753, *P* < .001) in triglycerides after leptin treatment. There was no significant correlation between baseline HbA1c and percent changes in triglycerides; however, baseline HbA1c tended to correlate with absolute change in triglycerides (*r* = -.405, *P* = .085). Levels of apolipoproteins CII (Fig. 5B) and E (Fig. 5C), and ANGPTL3 (Fig. 5D) were correlated with percent changes in triglycerides. Baseline apolipoprotein CIII levels tended to correlate with percent changes in triglycerides (*r* = -.447, *P* = .055). Baseline levels of apolipoproteins CII (*r* = -.781, *P* < .001), CIII (*r* = -.682, *P* = .001), and E (*r* = -.813, *P* < .001), and ANGPTL3 (*r* = -.593, *P* = .008) were correlated with numeric changes in triglycerides.

Baseline triglycerides were correlated with changes in HbA1c (Fig. 5E); however, there was no significant correlation between baseline HbA1c and change in HbA1c after exogenous leptin treatment. Baseline levels of apolipoproteins CII (Fig. 5F), CIII (Fig. 5G), and E (Fig. 5H), and ANGPTL3 (Fig. 5I) were correlated with changes in HbA1c and GLP-1 (*r* = -.495, *P* = .031; *r* = -.474, *P* = .040; *r* = -.539, *P* = .017; *r* = -.567, *P* = .011; respectively). Baseline apolipoprotein B levels tended to correlate with changes in NAS (*r* = -.432, *P* = .074) and NASH score (*r* = -.406, *P* = .095). Baseline apolipoprotein CII and CIII levels also tended to correlate with changes in hepatic fat (*r* = -.441, *P* = .058; *r* = -.447, *P* = .055; respectively).

### Hepatic Response based on NASH Score

Nine of 18 subjects with paired liver biopsies (50%) were classified as hepatic responders, defined as those who had a NASH score decrease of ≥2 points baseline, without an increase in fibrosis score by >1 point. When baseline characteristics of hepatic responders and nonresponders were compared, no significant difference was found in gender, age, the presence of pathologic variants of the *LMNA* gene, NASH and NAS score, BMI, total tissue %fat, HbA1c, and fasting triglycerides. The difference in baseline leptin level did not reach statistical significance but trended lower in the responder group compared to nonresponders (Table 4). Baseline levels of apolipoproteins, ANGPTL3, ANGPTL4, GIP, GLP-1, ghrelin, peptide YY, ANGPTL4, IGFBPs, fibroblast growth factor-21 (FGF21), IL-1, IL-6, IL-10, monocyte chemoattractant protein-1, adiponectin, and growth differentiation factor-15 (GDF15) were comparable. Although patients with hepatic response had slightly lower baseline IGF-1 levels, this difference was not significant between hepatic responders and nonresponders. Baseline tumor necrosis factor-alpha (TNF-α) trended lower in the hepatic responders compared to nonresponders.

**Table 4.** Comparison of baseline characteristics of responders and nonresponders to exogenous leptin classified based on response with improvement in liver histology (a decrease in the total NASH score of ≥2 without an increase in fibrosis)

	Hepatic responders (n = 9)	Hepatic nonresponders (n = 9)	*P* value
Age (y)	41 ± 18	46 ± 18	.600
Gender (M:F)	1:8	2:7	1.000
Pathologic *LMNA* variant (n, %)	3 (33%)	2 (22%)	1.000
BMI (kg/m^2^)	25.0 ± 4.6	28.6 ± 6.8	.211
Body weight (kg)	70.3 ± 18.8	82.3 ± 24.9	.267
Waist/hip ratio	0.96 ± 0.07	1.03 ± 0.08	.072
FMR	1.58 ± 0.34	1.72 ± 0.28	.349
NASH score	6 ± 2	6 ± 1	.779
NAS	5 ± 1	5 ± 1	.659
Liver fat (Dixon MR method) (%)	12.66 ± 4.23	13.90 ± 8.53	.702
Hemoglobin A1c (%)	8.7 ± 2.3	9.3 ± 1.1	.459
Triglyceride (mg/dL)*^a^*	1307 ± 2201	1291 ± 1710	.991
ALT (IU/L)*^a^*	46 ± 26	60 ± 38	.365
AST (IU/L)*^a^*	33 ± 14	53 ± 38	.236
Carbohydrate intake (g/day)	215 ± 41	157 ± 43	**.012**
REE (kcal)	1789 ± 286	1921 ± 397	.428
Fasting leptin level (ng/mL)	14.5 ± 8.6	25.1 ± 12.8	.056
Apolipoprotein B (mg/dL)	154 ± 24	125 ± 42	.095
Apolipoprotein CII (mg/dL)*^a^*	44 ± 43	38 ± 38	.703
Apolipoprotein CIII (mg/dL)*^a^*	105 ± 87	103 ± 90	.736
Apolipoprotein E (mg/dL)*^a^*	15 ± 15	15 ± 16	.868
Apolipoprotein AI (mg/dL)	42 ± 10	48 ± 25	.509
Apolipoprotein AII (mg/dL)	29 ± 5	32 ± 4	.154
ANGPTL3 (ng/mL)	15 ± 8	13 ± 6	.647
ANGPTL4 (ng/mL)	141 ± 68	160 ± 52	.510
IGF-1 (ng/mL)	123 ± 63	131 ± 79	.827
IGFBP-1 (ng/mL)*^a^*	2.79 ± 2.37	4.02 ± 4.98	.864
IGFBP-2 (ng/mL)	70.11 ± 15.45	69.03 ± 12.82	.874
IGFBP-3 (ng/mL)	2736.33 ± 547.72	2828.44 ± 928.18	.801
GIP (pg/mL)	126.19 ± 97.47	184.00 ± 162.93	.375
Ghrelin (pg/mL)	91.89 ± 61.15	71.49 ± 61.95	.492
GLP-1 (pg/mL)	30.76 ± 50.50	18.67 ± 16.18	.504
Peptide YY (pg/mL)*^a^*	131.37 ± 179.47	149.86 ± 204.60	.409
IL-1β (pg/mL)*^a^*	1.01 ± 0.75	7.98 ± 21.79	.585
IL-6 (pg/mL)*^a^*	16.85 ± 28.80	18.42 ± 48.31	.232
IL-10 (pg/mL)*^a^*	4.30 ± 2.67	7.89 ± 14.36	.905
TNF-α (pg/mL)*^a^*	7.32 ± 3.42	11.54 ± 6.94	.059
MCP1 (pg/mL)*^a^*	596.89 ± 180.08	656.51 ± 383.49	.679
FGF21 (pg/mL)*^a^*	446.88 ± 1043.95	206.97 ± 170.53	.293
GDF15 (pg/mL)*^a^*	1180.89 ± 764.71	1115.44 ± 410.71	.824
Adiponectin (ng/mL)	3354.43 ± 1678.95	3248.61 ± 1538.35	.891

Categorical parameters are compared using the chi-square test. An independent sample *t*-test is used to compare continuous variables. Bold value denote statistical significance at the *P* < .05 level.

Abbreviations: ALT, alanine aminotransferase; ANGPTL3, angiopoietin-like protein 3; ANGPTL4, angiopoietin-like protein 4; AST, aspartate aminotransferase; BMI, body mass index; FGF21, fibroblast growth factor 21; FMR, fat mass ratio (%fat trunk/%fat legs); GDF15, growth differentiation factor 15; GIP, glucose-dependent insulinotropic polypeptide; GLP-1, glucagon-like peptide-1; IGF, insulin-like growth factor; IGFBP, IGF-binding protein; IL, interleukin; *LMNA*, lamin A/C; MCP1, monocyte chemoattractant protein-1; REE, resting energy expenditure; NASH score, the sum of scores for steatosis, lobular inflammation, ballooning and fibrosis; NAS, nonalcoholic fatty liver disease activity score, the sum of scores for steatosis, lobular inflammation, and ballooning; TNF-α, tumor necrosis factor-alpha.

^a^Tests are run on log-transformed data. Data are presented as mean ± standard deviation (SD) or frequency. Carbohydrate intake was evaluated in 8 subjects in the hepatic responders group.

Interestingly, responders had a significantly higher baseline carbohydrate intake compared to nonresponders (Table 4). Of the parameters evaluated with ROC analysis to predict hepatic response, the AUC of baseline carbohydrate intake was the strongest predictor of response (Table 5). There was a mean decrease of 11 ± 69 g in carbohydrate intake after 1 year of treatment with metreleptin. This change was -29 ± 77 g in hepatic responders compared to 5 ± 60 g in hepatic nonresponders; however, the difference was statistically not significant (*P* = .114). The predictive value for leptin levels also reached statistical significance. The optimal cut-point estimated for baseline leptin to predict liver response was 16.6 ng/dL (Youden's index), which had moderate sensitivity and specificity (78% each). Baseline IGF-1 levels were not a significant predictor of hepatic response. However, patients with hepatic response had a greater increase in IGF-1 at month 3 although this difference was not statistically significant (41 ± 34 ng/dL vs 17 ± 43 ng/dL, *P* = .204; ROC AUC: 0.706 [95% CI, 0.461-0.950], *P* = .131). Baseline triglyceride level, NASH score, NAS, and hepatic fat % by MRI did not predict responder status (Table 5). There was a negative correlation between adiponectin and liver enzymes ALT (*r* = -.580, *P* = .009) and AST (*r* = -.630, *P* = .004).

**Table 5.** Predictors of hepatic response to metreleptin, ROC analysis

	AUC (95% CI)	*P* value
Carbohydrate intake (g/day)	0.819 (0.612-1.000)	**.027**
Leptin (ng/mL)	0.778 (0.552-1.000)	**.047**
Triglyceride (mg/dL)	0.556 (0.268-0.843)	.691
NAS	0.562 (0.287-0.836)	.659
NASH score	0.543 (0.261-0.825)	.757

ROC analysis is used to determine potential predictors of metreleptin response. Data are presented as mean ± standard deviation (SD). Triglycerides are reported as geometric mean and geometric SD factor. Carbohydrate intake was evaluated in 8 patients in the responders group. Bold values denote statistical significance at the *P* < .05 level.

Abbreviations: AUC, area under the curve; ROC, receiver operating characteristic.

REE tended to decrease more in patients with hepatic response compared to nonresponders (198 ± 312 kcal vs 49 ± 85 kcal decrease at month 12; *P* = .069). A high degree of variability was noted in the REE of the responder group and 1 pediatric subject had an increase in REE over the 12 months while the other 8 participants had decreased REE. A linear relationship between REE and changes in carbohydrate intake was noted (*r* = .750; *P* < .001). This relationship was significant in hepatic responders (*r* = .885; *P* = .004) but not in nonresponders (*r* = .393; *P* = .296).

### Metabolic Response to Metreleptin

Of the 19 subjects, 6 exhibited a reduction in HbA1c >1% at month 12. A triglyceride reduction of more than 30% was observed in 9 subjects. Overall, 9 of 19 subjects were considered to have a significant metabolic response to metreleptin, based on a glycemic response, lipid response, and hepatic response (meeting at least 2 of 3 criteria at month 12). We did not exclude the patient who did not undergo a second liver biopsy from this analysis, as this individual showed neither an HbA1c nor a triglyceride response and was thus automatically categorized as a “nonresponder,” despite the lack of paired liver biopsy data.

Baseline characteristics of patients with a metabolic response based on the criteria described previously and others were presented in Table 6. There was no significant difference in gender, age, and the presence of pathologic variants of the *LMNA* gene. Patients with metabolic response had slightly lower body weight, BMI, waist/hip ratio, FMR, and higher REE at baseline; however, none of these differences were statistically significant. On the other hand, baseline leptin was significantly lower (range: 5.9-23.9 ng/mL). Baseline fasting triglycerides were significantly higher in patients with metabolic response. All metabolic responders had triglyceride levels >200 mg/dL (range: 211-7004 mg/dL), though levels varied among individuals. Although they had slightly higher NAS, hepatic fat, ALT, AST, and HbA1c, these differences were not significant. Patients with metabolic response had lower baseline IGF-1, and higher apolipoprotein CII, apolipoprotein CIII, apolipoprotein E, and ANGPTL3 levels. Baseline levels of GIP, GLP-1, ghrelin, peptide YY, ANGPTL4, IGFBPs, FGF21, IL-1, IL-6, IL-10, TNF-α, and monocyte chemoattractant protein-1 were similar. Adiponectin levels tended to be decreased. Also, baseline GDF15 trended higher.

**Table 6.** Comparison of baseline characteristics of patients with PL to exogenous leptin classified based on response with improvements in (1) HbA1c (more than 1% HbA1c reduction from baseline), (2) triglycerides (more than 30% decrease in triglycerides from baseline), and (3) liver histology (a decrease in the total NASH score of ≥2 without an increase in fibrosis)

Parameters	Patients with ≥2 metabolic response criteria (n = 9)	Patients with <2 metabolic response criteria (n = 10)	*P* value
Age (y)	41 ± 14	45 ± 20	.695
Gender (M:F)	8:1	8:2	1.000
Pathologic *LMNA* variant (n, %)	3 (33%)	3 (30%)	1.000
BMI (kg/m^2^)	25.8 ± 5.4	27.7 ± 6.2	.488
Body weight (kg)	73.3 ± 20.6	77.6 ± 23.9	.680
Waist/hip ratio	0.99 ± 0.10	1.01 ± 0.06	.596
FMR	1.64 ± 0.31	1.87 ± 0.69	.381
NASH score	6 ± 2	6 ± 2	.319
NAS	5 ± 1	4 ± 1	.100
Liver fat (Dixon MR method) (%)	14.3± 7.5	11.5 ± 6.0	.375
Hemoglobin A1c (%)	9.3 ± 1.6	8.4 ± 2.0	.273
Triglyceride (mg/dL)*^a^*	2171 ± 2407	410 ± 483	**.008**
ALT (IU/L)*^a^*	54 ± 29	49 ± 37	.610
AST (IU/L)*^a^*	49 ± 35	35 ± 22	.307
Carbohydrate intake (g/day)	181 ± 43	186 ± 55	.853
REE (kcal)	1919 ± 384	1789 ± 287	.413
Leptin (ng/mL)	13.5 ± 5.5	27.6 ± 16.2	**.024**
Apolipoprotein B (mg/dL)	154 ± 32	132 ± 41	.212
Apolipoprotein CII (mg/dL)*^a^*	61 ± 47	21 ± 15	**.011**
Apolipoprotein CIII (mg/dL)*^a^*	148 ± 64	61 ± 49	**.015**
Apolipoprotein E (mg/dL)*^a^*	23 ± 18	7 ± 4	**.002**
Apolipoprotein AI (mg/dL)	46 ± 25	46 ± 13	.966
Apolipoprotein AII (mg/dL)	30 ± 5	32 ± 4	.543
ANGPTL3 (ng/mL)	17 ± 7	11 ± 5	**.040**
ANGPTL4 (ng/mL)	158 ± 65	147 ± 55	.709
IGF-1 (ng/mL)	105 ± 58	150 ± 71	**.036**
IGFBP-1 (ng/mL)*^a^*	4.22 ± 4.93	2.56 ± 2.19	.483
IGFBP-2 (ng/mL)	71.94 ± 17.31	67.59 ± 9.10	.495
IGFBP-3 (ng/mL)	2711.00 ± 808.56	2786.40 ± 700.56	.830
GIP (pg/mL)	137.79 ± 77.63	166.86 ± 167.52	.640
Ghrelin (pg/mL)	77.52 ± 64.94	83.17 ± 56.85	.842
GLP-1 (pg/mL)	32.12 ± 49.68	19.07 ± 17.31	.445
Peptide YY (pg/mL)*^a^*	116.38 ± 165.12	152.49 ± 205.25	.989
IL-1β (pg/mL)*^a^*	1.01 ± 0.72	7.25 ± 20.68	.701
IL-6 (pg/mL)*^a^*	7.64 ± 8.74	24.98 ± 51.15	.721
IL-10 (pg/mL)*^a^*	3.36 ± 1.95	8.13 ± 13.51	.355
TNF-α (pg/mL)*^a^*	8.33 ± 3.79	9.87 ± 7.16	.659
MCP1 (pg/mL)	675.22 ± 321.27	601.96 ± 265.58	.594
FGF-21 (pg/mL)*^a^*	468.91 ± 1034.42	176.59 ± 179.45	.958
GDF15 (pg/mL)	1383.78 ± 613.58	862.10 ± 501.66	.057
Adiponectin (ng/mL)	2725.72 ± 1466.04	3950.19 ± 1447.20	.085

Categorical parameters are compared using the chi-square test. An independent sample *t*-test is used to compare continuous variables. Bold values denote statistical significance at the *P* < .05 level.

Abbreviations: ALT, alanine aminotransferase; ANGPTL3, angiopoietin-like protein 3; ANGPTL4, angiopoietin-like protein 4; AST, aspartate aminotransferase; BMI, body mass index; FGF21, fibroblast growth factor 21; FMR, fat mass ratio (%fat trunk/ %fat legs); GDF15, growth differentiation factor 15; GDP15, growth /differentiation factor 15; GIP, glucose-dependent insulinotropic polypeptide; GLP-1, glucagon-like peptide-1; IGF, insulin-like growth factor; IGFBP, IGF-binding protein; IL, interleukin; *LMNA*, lamin A/C; MCP1, monocyte chemoattractant protein-1; NASH score, the sum of scores for steatosis, lobular inflammation, ballooning, and fibrosis; NAS, nonalcoholic fatty liver disease activity score, the sum of scores for steatosis, lobular inflammation, and ballooning; REE, resting energy expenditure; TNF-α, tumor necrosis factor-alpha.

^a^Tests are run on log-transformed data. Data are presented as mean ± standard deviation (SD) or frequency. Carbohydrate intake was evaluated in 8 subjects in the metabolic responders group.

Of the parameters evaluated with ROC analysis to predict metabolic response, the AUC values of baseline triglycerides, and apolipoproteins CII, CIII, and E were statistically significant (Table 7A). The predictive value for baseline leptin levels also tended to reach significant levels. Baseline IGF-1 level was not a significant predictor of metabolic response (*P* > .05); however, ROC analysis showed an AUC of 0.789 (95% CI, 0.570-1.000; *P* = .034) for the change in IGF-1 at month 12 and 0.844 (95% CI, 0.668-1.000; *P* = .011) for the change in IGF-1 percentile at month 12.

**Table 7.** Predictors of metabolic response to exogenous leptin, ROC analysis

n = 19	AUC (95% CI)	*P* value
*Baseline*		
Leptin (ng/mL)	0.532-0.979	.060
Triglycerides (mg/dL)	0.677-1.000	**.009**
NAS score	0.460-0.940	.142
NASH score	0.390-0.899	.288
Apolipoprotein B (mg/dL)	0.440-0.960	.142
Apolipoprotein CII (mg/dL)	0.663-1.000	**.011**
Apolipoprotein CIII (mg/dL)	0.580-0.998	**.034**
Apolipoprotein E (mg/dL)	0.763-1.000	**.003**
ANGPTL3 (mg/dL)	0.506-0.983	.072
*At month 3*		
% Decrease in triglycerides	0.700-1.000	**.007**
% Decrease in apolipoprotein B	0.506-1.000	**.041**
% Decrease in apolipoprotein CII	0.770-1.000	**.003**
% Decrease in apolipoprotein CIII	0.663-1.000	**.011**
% Decrease in apolipoprotein E	0.731-1.000	**.004**
% Decrease in ANGPTL3	0.645-1.000	**.011**

Bold values denote statistical significance at the *P* < .05 level.

Abbreviations: ANGPTL3, angiopoietin-like protein 3; AUC, area under the curve; NASH score, the sum of scores for steatosis, lobular inflammation, ballooning, and fibrosis; NAS, nonalcoholic fatty liver disease activity score, the sum of scores for steatosis, lobular inflammation, and ballooning.

The Youden's index identified optimal cut-points for baseline triglycerides (baseline triglyceride ≥327 mg/dL; sensitivity: 89%; 95% CI, 52-100; specificity: 80%; 95% CI, 44-98) and apolipoprotein E (baseline Apo E ≥87 mg/dL; sensitivity: 78%; 95% CI, 40-97; specificity: 90%; 95% CI, 56-100) to predict metabolic response. At month 3, percent reductions in triglycerides, apolipoproteins B, CII, CIII, and E, and ANGPTL3 were determined as significant predictors of metabolic response (Table 7B). Percent reductions in apolipoprotein E ≥24% at month 3 (sensitivity: 78%; 95% CI, 40-98; specificity: 100%; 95% CI, 69-100), apolipoprotein CII ≥9% at month 3 (sensitivity: 89%; 95% CI, 52-100; specificity: 90%; 95% CI, 56-100) were optimal cut-points to predict metabolic response at month 12.

### Correlation of Baseline Leptin Level With Metabolic Status and Changes in Apolipoprotein Levels

Table 3 shows correlation coefficients between leptin and baseline metabolic parameters. Leptin levels were positively correlated with BMI, waist/hip ratio, and high-density lipoprotein and LDL cholesterol; and negatively correlated with NAS and triglycerides. Leptin levels also tended to negatively correlate with baseline apolipoprotein CII, apolipoprotein CIII, apolipoprotein E, and positively correlate with apolipoprotein AII.

Baseline leptin levels were correlated with changes in FMR, and tended to correlate with reductions in HbA1c, NAS, and percent reductions in triglycerides. Leptin levels were correlated with percent changes in apolipoprotein B, apolipoprotein CII, apolipoprotein CIII, and apolipoprotein E after exogenous leptin treatment. Leptin was also correlated with early changes (percent changes at month 3) in triglycerides, apolipoprotein CII, apolipoprotein CIII, and apolipoprotein E (Table 8).

**Table 8.** Correlations between baseline leptin levels and changes after exogenous leptin therapy

	*r*	*P* value
Change in FMR	.531	**.019**
Change in HbA1c	.428	.067
Change in NAS	.430	.075
% Change in triglycerides	.399	.091
% Change in apolipoprotein B	.518	**.023**
% Change in apolipoprotein CII	.513	**.025**
% Change in apolipoprotein CIII	.551	**.014**
% Change in apolipoprotein E	.460	**.047**
% Change in triglycerides at month 3	.639	**.003**
% Change in apolipoprotein CII at month 3	.528	**.020**
% Change in apolipoprotein CIII at month 3	.524	**.021**
% Change in apolipoprotein E at month 3	.564	**.012**

Bold values denote statistical significance at the *P* < .05 level.

Abbreviations: FMR, fat mass ratio (%fat trunk/%fat legs); NAS, nonalcoholic fatty liver disease activity score, the sum of scores for steatosis, lobular inflammation, and ballooning.

### IGF-1 Levels Increase After Treatment With Exogenous Leptin and Correlate With Improvements in Liver Parameters

**Figure 6.**
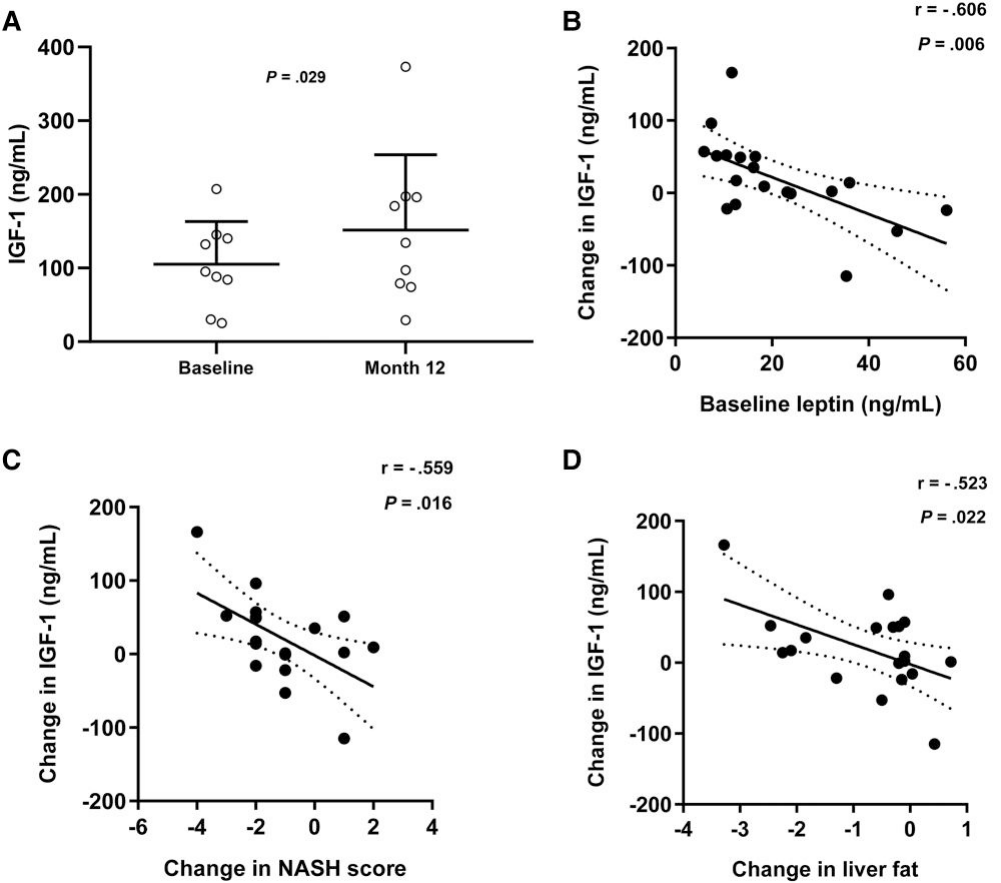
IGF-1 levels and correlation of changes in IGF-1 with baseline leptin and improvements in NASH score and liver fat. IGF-1 levels at baseline and after exogenous leptin in patients with a remarkable metabolic response (A). Correlation of changes in IGF-1 with baseline leptin (B), and changes in NASH score (C) and liver fat (D).

Table 9 shows the correlation between IGF-1 and IGFBPs and studied parameters. Baseline IGF-1 was negatively correlated with age and tended to correlate with HbA1c. At baseline, we did not detect significant correlations between IGF-1 and BMI, fat content on MRI, NAS, or NASH scores. Nine of 19 (47.4%) patients had baseline IGF-1 levels in the lowest quartile of the age- and gender-specific normal range. In patients with metabolic response, we observed a significant increase in IGF-1 levels at month 12 (Fig. 6A). There was a negative correlation between baseline leptin level and numeric IGF-1 increase (Fig. 6B). Change in IGF-1 was correlated with improvements in NAS (*r* = -.547; *P* = .019) and NASH score (Fig. 6C) and the reduction of hepatic steatosis measured by MRI (Fig. 6D).

**Table 9.** Correlation coefficients between IGF-1, IGFBPs, and the studied parameters

	*r*	*P* value
IGF-1		
Age	-.745	**<.001**
HbA1c	-.439	.060
IGFBP-1		
Apolipoprotein AI	.497	**.030**
ANGPTL4	.612	**.005**
Waist/hip ratio	.443	.057
GLP-1	.401	.088
FGF21	.413	.088
IGFBP-2		
NAS	.541	**.017**
NASH score	.480	**.037**
GDF15	.590	**.008**
GIP	.434	.064
IGFBP-3		
GIP	.537	**.018**
GDF15	.495	**.031**
Apolipoprotein AI	-.511	**.025**
Waist/hip ratio	-.440	.059
IL6	-.390	.099

Bold values denote statistical significance at the *P* < .05 level.

Abbreviations: ANGPTL4, angiopoietin-like protein 4; GDP15, growth /differentiation factor 15; GIP, glucose-dependent insulinotropic polypeptide; GLP-1, glucagon-like peptide-1; IGFBP, IGF-binding protein; NASH score, the sum of scores for steatosis, lobular inflammation, ballooning, and fibrosis; NAS, nonalcoholic fatty liver disease activity score, the sum of scores for steatosis, lobular inflammation, and ballooning.

At baseline, IGFBP-1 was positively correlated with apolipoprotein AI and ANGPTL4 and tended to correlate with waist/hip ratio, GLP-1, and FGF21. IGFBP-2 was positively correlated with NAS, NASH score, and GDF15, and tended to correlate with GIP. IGFBP-3 was positively correlated with GIP and GDF15 and negatively correlated with apolipoprotein AI. IGFBP-3 also tended to negatively correlate with waist-hip ratio and IL-6.

## Discussion

Our results provide support that leptin therapy can improve MASH and metabolic parameters in patients with PL; however, treatment response varies among individuals. Mixed-meal test completed on a subset of participants revealed significant reductions in triglyceride AUC and glucose excursions at several postmeal time points. Mixed-meal data taken together with frequent sampling data suggest that leptin therapy may cause attenuation of postmeal GIP peaks and cause increases in ghrelin levels, suggesting that there is crosstalk between leptin and gut hormone secretion. Exogenous leptin treatment is associated with reductions in levels of apolipoproteins associated with triglyceride metabolism. In addition, ANGPTL3 levels tended to decrease after leptin treatment. The metabolic and hepatic response to exogenous leptin treatment was associated with several factors, including the severity of metabolic disease at baseline, levels of triglycerides and triglyceride-rich apolipoproteins, baseline leptin levels, and changes in IGF-1 levels. Overall, we observed a significant increase in circulating IGF-1 levels at 3 months following leptin treatment. A significant rise in IGF-1 levels at 12 months was also noted in patients who showed a metabolic response. The change in IGF-1 levels correlated with the 12-month response in liver biopsies. Although not statistically significant, patients with a hepatic response exhibited a greater increase in IGF-1 at month 3. Moreover, metabolic changes that occur by 3 months appear to predict 12-month metabolic responses.

While the exact mechanism of dyslipidemia is not well understood in lipodystrophy, a common belief has always been that postmeal lipid clearance would be decreased in these subjects [30, 31]. Supporting this notion, previous data in HIV-associated lipodystrophy revealed that chylomicron-triacylglycerol-derived palmitate disposal was markedly lower in the 8-hour postmeal period [32]. In addition, insulin resistance due to excessive flux of fatty acids into liver can increase hepatic very LDL secretion, which cannot be disposed of due to reduced adipocyte mass [30]. Data from our mixed-meal test showed lower triglycerides, glucose, and FFA levels at several time points while under leptin treatment. Although not statistically significant, the reduction in energy/carbohydrate intake would be expected to reduce de novo lipogenesis and decrease triglyceride levels, especially in the fasting state [33]. In addition, we observed a decrease in FFAs while on leptin therapy, which was significant at several time points, although these reductions still appeared to be less than normal suppression of FFAs [34]. We also observed an attenuated GIP secretion in the mixed-meal test, following leptin treatment, consistent with our previous observation in a small cohort of men with MASH and relative leptin deficiency [18]. The decrease in GIP may reflect a reduction in glucose excursions postmeal [35]. Moreover, we observed an increase in circulating ghrelin 3 hours postmeal following leptin therapy. The primary role of GIP is to stimulate insulin secretion after meals. Its effect on satiety is complex [36]. On the other hand, the effect of ghrelin on satiety is well-established. Ghrelin is primarily secreted by the stomach when it is empty and acts on the hypothalamus to increase appetite and decrease satiety [37]. Recently, we and others have reported on the effects of GLP-1 agonists and tirzepatide, a GLP-1/GIP analogue, in patients with PL [38-40]. These limited observations point to the need for further investigation of the brain-gut-liver and adipocyte cross-talk in both normal and diseased states. Rodent studies suggest that leptin's central satiety effects may involve neurons that simultaneously express GLP-1 and leptin receptors. Combining leptin with GLP-1 activation has been shown to enhance appetite suppression, promote greater weight loss, and better preserve lean mass [41]. This highlights a potential synergy between incretin and leptin pathways, warranting further investigation. However, whether tirzepatide can serve as an effective adjuvant therapy to leptin in PL remains unknown at this time.

Exogenous leptin treatment has shown to improve certain metabolic aspects in patients with PL [42-46]; however, these effects are generally less robust and more variable compared to those observed in GL [15, 16, 44, 45, 47]. Our results, in general, are consistent with other studies in PL where individuals with milder metabolic abnormalities or higher leptin levels showed a lesser response to exogenous leptin treatment [17, 45]. In our study, we found that a baseline leptin level of 16.6 ng/dL could predict hepatic response. However, we do not believe that any leptin cut-off could reliably identify responders in the context of PL, and its implications for clinical practice are minimal, given the lack of standardization of leptin assays and the variability in concordance between assays. A recent analysis of 84 patients treated with exogenous leptin for 12 months, drawn from 3 studies conducted at the NIDDK and 2 studies conducted at the University of Michigan, revealed that patients with PL who exhibited a more favorable treatment response tended to have numerically lower leptin levels [48]. Real-world evidence also suggests a better metabolic response in patients with PL and lower leptin levels [49]. Despite these observations, neither the recent analysis from NIDDK/University of Michigan nor any other studies have identified a reliable cut-point to select responders to exogenous leptin. Leptin assays lack standardization, normative ranges have not been well established, results from common leptin assays are not interchangeable [48], and leptin levels may vary depending on factors such as age, gender, and BMI [48, 50].

We observed a significant reduction in triglyceride rich apolipoproteins CII, CIII, and E after leptin therapy and, in addition to baseline levels, early reductions of these levels predicted metabolic response at year 1. A recent analysis of long-term effectiveness of leptin therapy in patients with PL found more profound improvements in a subgroup of patients with HbA1c ≥6.5% or triglycerides ≥500 mg/dL, suggesting that baseline metabolic status might be important to predict the metabolic response to exogenous leptin in PL [17]. Strengthening this observation, significant reductions in HbA1c and fasting triglycerides were detected in a subgroup of patients with more severe baseline metabolic abnormalities (HbA1c ≥8.0% or triglycerides ≥500 mg/dL). The results of our current study support the concept that baseline metabolic disease severity plays a role in determining metabolic response in PL. Patients with a remarkable metabolic response exhibited higher triglycerides, triglyceride-rich apolipoproteins CII, CIII, and E, and ANGPTL3 levels, along with lower baseline leptin levels. Furthermore, baseline levels of apolipoproteins were found to correlate with subsequent changes following leptin treatment. ROC analysis revealed that baseline triglycerides and apolipoproteins CII, CIII, and E were significant predictors of metabolic response. Our previous work also suggested that reductions in HbA1c, fasting plasma glucose, and triglycerides were greater among patients with PL with higher baseline abnormalities [51].

We observed improvements in liver parameters following leptin treatment in this cohort of patient with PL who had variable leptin levels [18]. Half of our participants showed histological response with clinically relevant improvements in NASH score (and NAS) on biopsy after 1 year of treatment. Patients with a hepatic response had a significantly higher basal carbohydrate intake. Previous studies have shown that high-fat Western-style diet and high carbohydrate intake are associated with hepatic steatosis [52-54] and reduced carbohydrate consumption may improve liver fat metabolism [55]. In addition to baseline carbohydrate intake, fasting leptin may help predict hepatic response to exogenous leptin. In this current study, subjects who had lower leptin levels at baseline tended to have higher carbohydrate intake. How changes in carbohydrate intake are affected by exogenous leptin treatment and the effect on MASH deserves additional study. In a small study from 1997, Jenkins et al [56] showed that in an acute energy-restricted setting, the changes in serum leptin and carbohydrate intake were closely related while a similar correlation was not observed with fat intake. In addition, Domingos et al [57] reported through elegant optogenetic experiments that leptin suppresses the ability of sucrose to drive taste-independent dopamine neuronal activation. A recent study, on the other hand, concluded that exogenous leptin can improve insulin sensitivity and decrease hepatic triglycerides while carefully controlling caloric intake and diet composition [58]. Metz et al [59] later confirmed that leptin exhibits antisteatotic effects independent of food intake by demonstrating its direct modulation of hepatic triglyceride export following an acute leptin injection. Furthermore, they found that leptin fails to promote hepatic triglyceride export in humans lacking intact hepatic autonomic innervation, suggesting that the effects of leptin on liver metabolism in humans are mediated via the autonomic nervous system. Although these findings highlight a complex relationship of leptin to carbohydrate intake, it is still intriguing to speculate that the patients with PL and lower leptin levels derive a higher value of food reward from carbohydrates and thus demonstrate higher carbohydrate intake. In addition, after leptin therapy, we observed a significant reduction in GIP and a numerical decrease in GLP-1 levels measured at 60 minutes during the mixed-meal test. At the nucleus tractus solitarius, leptin receptor-expressing neurons receive signals from the gut that synergize with leptin action, triggering the production of the food intake-suppressing cleavage product GLP-1 from preproglucagon. While preproglucagon/GLP-1-mediated neurotransmission contributes to appetite-suppressing effects of GLP-1NTS cells, recent research suggests that additional pathways engaged by leptin receptor-expressing nucleus tractus solitarius cells are dominant in the suppression of food intake [41]. Since leptin can directly correct this altered value attribution through specific neural circuits, those patients may have a better response to exogenous leptin therapy.

Interestingly, nearly half of the patients exhibited baseline IGF-1 levels in the lowest quartile of the age- and gender-specific normal range, which increased significantly 3 months after leptin therapy. Furthermore, patients with metabolic response showed more severely suppressed baseline IGF-1 levels. The rise in IGF-1 levels after leptin therapy was found to correspond with improvements in NAS, NASH score, and hepatic fat content, suggesting IGF-1's potential as a biomarker for monitoring the effectiveness of leptin replacement therapy in individuals with PL. Prior studies have consistently reported low levels of IGF-1 in insulin-resistant conditions, such as MASLD, indicating a potential link between hepatic insulin resistance and IGF-1 regulation, possibly through modulation of GH-stimulated synthesis of hepatic IGF-1 [60-63]. Hepatic IGF-1 mRNA was notably lower in individuals with higher steatosis and NAS, inversely correlating with glucose parameters [64]. Additionally, low serum IGF-1 levels have been associated with increased MASLD severity, suggesting its utility as a marker for monitoring disease progression or improvements postintervention [65]. Low levels of IGF-1 have previously been reported in patients with FPLD and other forms of lipodystrophy [66, 67]. Another possible explanation is that low leptin levels suppress the GH/IGF-1 axis. Leptin plays a role in the neuroendocrine regulation of pulsatile GH secretion. Leptin receptors are expressed in hypothalamic nuclei involved in GH regulation. Previous studies have demonstrated leptin's potent stimulatory effect on pulsatile GH secretion and GH response to GHRH [68].

Leptin substitution therapy has shown promise in increasing IGF-1 levels in absolute leptin-deficient states such as congenital leptin deficiency [69]. Additionally, the use of recombinant human IGF-1 (rhIGF-1) was associated with improvements in acanthosis nigricans, and levels of plasma glucose, insulin, fructosamine and HbA1c in patients with extreme insulin resistance syndromes, including congenital generalized lipodystrophy (CGL) [70]. Two subjects with CGL maintained normal glucose tolerance and experienced a significant reduction in glucose and triglyceride levels after rhIGF-1 treatment [71]. In a later report, rhIGF-1 treatment was found to be effective in lowering glucose and triglyceride levels over the long term in a CGL patient; however, it was difficult to suppress the patient's voracious appetite [72]. Furthermore, interventions targeting IGF-1 levels, such as tesamorelin and GH, have exhibited benefits in improving liver histology and reducing liver fat in patients with MASLD [73, 74]. IGF-I exerts a strong antifibrotic effect, acting both directly through the GH/IGF system and by regulating hepatoprotective genes [25]. Also, low IGF-I levels may be due to the reduced synthetic capacity of the liver, combined with a decrease in GH receptors, as previously shown in patients with chronic liver disease, which correlates with indicators of liver function [75]. In this context, 1 possible explanation for the increased IGF-1 levels in hepatic responders could be an improvement in liver metabolism, leading to better GH responsiveness of the liver and enhancing its ability to secrete IGF-1 by improving its synthesis capabilities.

Our findings, in conjunction with existing literature, suggest that monitoring IGF-1 levels may offer additional insights into the effectiveness of leptin replacement therapy in managing lipodystrophy and related metabolic complications. Taking our observations together with other data that are reviewed previously, it is possible to propose that those PL patients with lower leptin levels may have a simultaneous defect in regulating carbohydrate intake which may drive hepatic insulin resistance with a read-out of a lower IGF-1 levels. We believe that leptin therapy in these selected individuals may address these abnormalities and improving hepatic de novo lipogenesis with a readout of improved fasting hypertriglyceridemia and liver steatosis (potentially hepatocyte injury and inflammation).

Limitations of our study include the open-label design and small sample size despite being the most comprehensive and systematic evaluation of liver histopathology and mixed-meal investigations after leptin therapy in patients with PL. In addition, the participants were quite heterogeneous in age, comorbidities, and medication usage, which, in addition to differences in baseline leptin levels, likely contributes to the heterogeneity in response. Also, patients were advised to follow a diet with a balanced macronutrient composition and to limit energy and fat intake to better manage their diabetes and high triglycerides, in line with prestudy dietary recommendations, but no strict dietary monitoring was enforced. Both high carbohydrate intake at baseline and low leptin levels were associated with response to exogenous leptin therapy. However, whether high carbohydrate intake serves as a confounder or a mediator in the relationship between low leptin levels and metreleptin response remains undetermined. Since the different analytes were investigated to provide a comprehensive evaluation of leptin therapy to integrate biochemical and hormonal changes with therapy and describe a comprehensive signature of leptin response, correction for multiple comparisons was not undertaken. We should also note that the metreleptin dose was increased in some participants during the study period. As a result, the dose used at 2 time points for the frequent sampling substudy was different, with the month 12 dose being higher than the starting dose in some subjects. We should finally note that all parameters investigated were determined a priori before the study was initiated and all endpoints were part of the study design as exploratory analyses.

In conclusion, exogenous leptin therapy can improve MASH and metabolic status in patients with PL; however, treatment response is heterogeneous. Leptin therapy causes primarily a reduction in fasting triglyceride levels while postmeal excursions remain relatively stable. Following leptin therapy, the GIP response is attenuated, and late ghrelin peaks appear to be more robust, suggesting crosstalk between leptin and gut hormones. Moreover, leptin therapy lowers levels of apolipoproteins associated with triglyceride metabolism. Several factors appear to be related to the hepatic and/or metabolic response to exogenous leptin treatment, including the severity of metabolic disease at baseline, levels of triglycerides and triglyceride-rich apolipoproteins, baseline leptin levels, and changes in IGF-1 levels. Future efforts will be needed to clarify the role of these different parameters in predicting response of patients to exogenous leptin in the treatment of MASH and other metabolic abnormalities.

## Data Availability

Some or all datasets generated and/or analyzed during the current study are not publicly available but are available from the corresponding author on reasonable request.

## Clinical Trial Registration

NCT01679197 (UM IRBMED: HUM00058708).

## Abbreviations

ANGPTL: angiopoietin-like protein
AUC: area under the curve
BMI: body mass index
CGL: congenital generalized lipodystrophy
DEXA: dual-energy X-ray absorptiometry
FFA: free fatty acid
FGF: fibroblast growth factor
FGF21: fibroblast growth factor 21
FMR: fat mass ratio
FPLD: familial partial lipodystrophy
GDF15: growth differentiation factor-15
GIP: glucose-dependent insulinotropic polypeptide
GL: generalized lipodystrophy
GLP-1: glucagon-like peptide-1
HbA1c: glycated hemoglobin
IGF: insulin-like growth factor
IGFBP: IGF-binding protein
IL: interleukin
LDL: low-density lipoprotein
MASH: metabolic disease-associated steatohepatitis
MASLD: metabolic dysfunction-associated steatotic liver disease
MCP1: monocyte chemoattractant protein-1
MRI: magnetic resonance imaging
NAS: nonalcoholic fatty liver disease activity score
NASH: nonalcoholic steatohepatitis
PL: partial lipodystrophy
REE: resting energy expenditure
rhIGF-1: recombinant human IGF-1
ROC: receiver operating characteristic
TNF-α: tumor necrosis factor-alpha
